# Confirmation of *GRHL2* as the gene for the DFNA28 locus

**DOI:** 10.1002/ajmg.a.36017

**Published:** 2013-06-27

**Authors:** Barbara Vona, Indrajit Nanda, Cordula Neuner, Tobias Müller, Thomas Haaf

**Affiliations:** 1 Institute of Human Genetics, Julius Maximilians UniversityWürzburg, Germany; 2 Department of Bioinformatics, Julius Maximilians UniversityWürzburg, Germany

**Keywords:** autosomal dominant hearing impairment, DFNA28, *GRHL2*, haploinsufficiency, postlingual hearing impairment, progressive hearing loss

## Abstract

More than 10 years ago, a c.1609_1610insC mutation in the grainyhead-like 2 (*GRHL2*) gene was identified in a large family with nonsyndromic sensorineural hearing loss, so far presenting the only evidence for *GRHL2* being an autosomal-dominant deafness gene (DFNA28). Here, we report on a second large family, in which post-lingual hearing loss with a highly variable age of onset and progression segregated with a heterozygous non-classical splice site mutation in *GRHL2*. The c.1258-1G>A mutation disrupts the acceptor recognition sequence of intron 9, creating a new AG splice site, which is shifted by only one nucleotide in the 3′ direction. cDNA analysis confirmed a p.Gly420Glufs*111 frameshift mutation in exon 10. © 2013 Wiley Periodicals, Inc.

## INTRODUCTION

Autosomal dominant nonsyndromic hearing impairment accounts for approximately 20% of hereditary hearing loss. To date, there are 54 autosomal dominant loci with 27 associated causative genes identified [Van Camp and Smith, 2012]. The DFNA28 (OMIM: 608641) locus is comprised of *GRHL2* (OMIM: 608576) with the alias *TFCP2L3* (transcription factor cellular promoter 2-like 3), which is a widely expressed transcription factor in human epithelial tissues [Werth et al., 2010]. *GRHL2* spans approximately 177 kb on chromosome 8q22.3 (NCBI 37/hg19) and contains 16 exons, which translate into a 625 amino acid protein. It was first associated with the DFNA28 locus through mapping studies involving a five-generation North American family affected with mild to moderate post-lingual progressive bilateral sensorineural hearing loss. In this family, affected members had a heterozygous c.1609_1610insC mutation in exon 13 [Peters et al., 2002]. In addition, several single nucleotide polymorphisms (SNPs) in *GRHL2* have been associated with marginal significance with age-related hearing impairment susceptibility [Van Laer et al., 2008]. Considering that a second disease-causing mutation has not been reported, one might begin to suspect that *GRHL2* is not a bonafide deafness gene.

The expression and function of *GRHL2* have previously been investigated in animal studies. Northern blot and in situ hybridization studies in the mouse demonstrated high *Grhl2* expression in the cochlear duct at embryonic day 18.5 and postnatal day 5 [Peters et al., 2002; Wilanowski et al., 2002]. *Grhl2*^−/−^ knockout mice were embryonic lethal, displaying split face and neural tube defects. *Grhl2*^+/−^/*Grhl3*^+/−^ compound heterozygotes were viable and exhibited neural tube defects of varying severity. Evidently, coordinated expression of GRHL transcription factors in the non-neural ectoderm is important for neural tube closure [Rifat et al., 2010]. Unfortunately, hearing was not tested in heterozygous animals. *Tol2* transposon-mediated insertional mutagenesis in zebrafish produced offspring with enlarged otocysts, reduced or absent otoliths, malformed semicircular canals, insensitivities to sound stimulation, and abnormal swimming position despite the normal appearance of hair cells in the inner ear. Upon wild type human *GRHL2* mRNA injection, the inner ear defects in the zebrafish were rescued, whereas injection with mutant human *GRHL2* was unable to rescue otic defects [Han et al., 2011]. This suggests a conserved structure and function of GRHL2 in vertebrate inner ear development.

## MATERIALS AND METHODS

The study was approved by the Ethics Committee of the University of Würzburg.

### Mutation Analysis

Genomic DNA was extracted from whole blood using a standard salt extraction method, and was submitted to Otogenetics Corporation (Norcross, GA) for exome capture (targeting 80 known deafness genes) and next generation sequencing (NGS) on a HiSeq2000 (Illumina, San Diego, CA). Paired-end reads of 90–100 bp were analyzed for quality, exome coverage, and exome-wide SNP/InDels using the platform provided by DNAnexus (Mountain View, CA), to which we applied our systematic analysis beginning with the removal of calls that did not meet certain quality and confidence thresholds. Intronic variants not predicted to affect splicing or regulation were also removed, since they are not likely to impact protein structure and function. As we expected the causative dominant mutation to be absent in the healthy population, it is unlikely to be reported in variant databases such as dbSNP and SwissVar. We also used SIFT [Ng and Henikoff, 2001], PolyPhen-2 [Adzhubei et al., 2010], and MutationTaster [Schwarz et al., 2010] to predict the impact of any identified amino acid substitution on the protein structure and function and to predict disease causing potential resulting from sequence alterations.

To validate the identified mutation, an amplicon containing the *GRHL2* c.1258-1G>A mutation was PCR amplified from genomic DNA using standard PCR cycling conditions with forward primer 5′-GGATTTCACTGGTTTAGGG-3′ and reverse primer 5′-AGCGTAGACTTCAAGTGAGC-3′ (Metabion, Martinsried, Germany). PCR products were sequenced with an ABI 3130xl 16-capillary sequencer (Life Technologies, Carlsbad, CA).

### RNA Analysis

RNA samples were isolated from saliva using a standard protocol from the Oragene RNA collection kit (DNA Genotek, Ottawa, ON, Canada). RNA quality and quantity were assessed with a NanoDrop spectrophotometer (NanoDrop Technologies, Wilmington, DE). cDNA was produced using the SuperScript III First-Strand Synthesis SuperMix RT-PCR kit (Invitrogen, Karlsruhe, Germany). The *GRHL2* region of interest was amplified using standard PCR cycling conditions from the synthesized cDNA with forward primer 5′-GGAAATACTGGCACTCTCG-3′ and reverse primer 5′-ACCTTCTCGTTCATCATCC-3′. A second round nested PCR continued with inner forward primer 5′-CCGTGAATTGCTTGAGCACA-3′ and reverse primer 5′-GGTTTGCAAAGTGAACATCAG-3′ in order to shorten the amplicon length for Sanger sequencing and enhance the product on the agarose gel.

## RESULTS

### Pedigree Analysis

**Figure 1:**
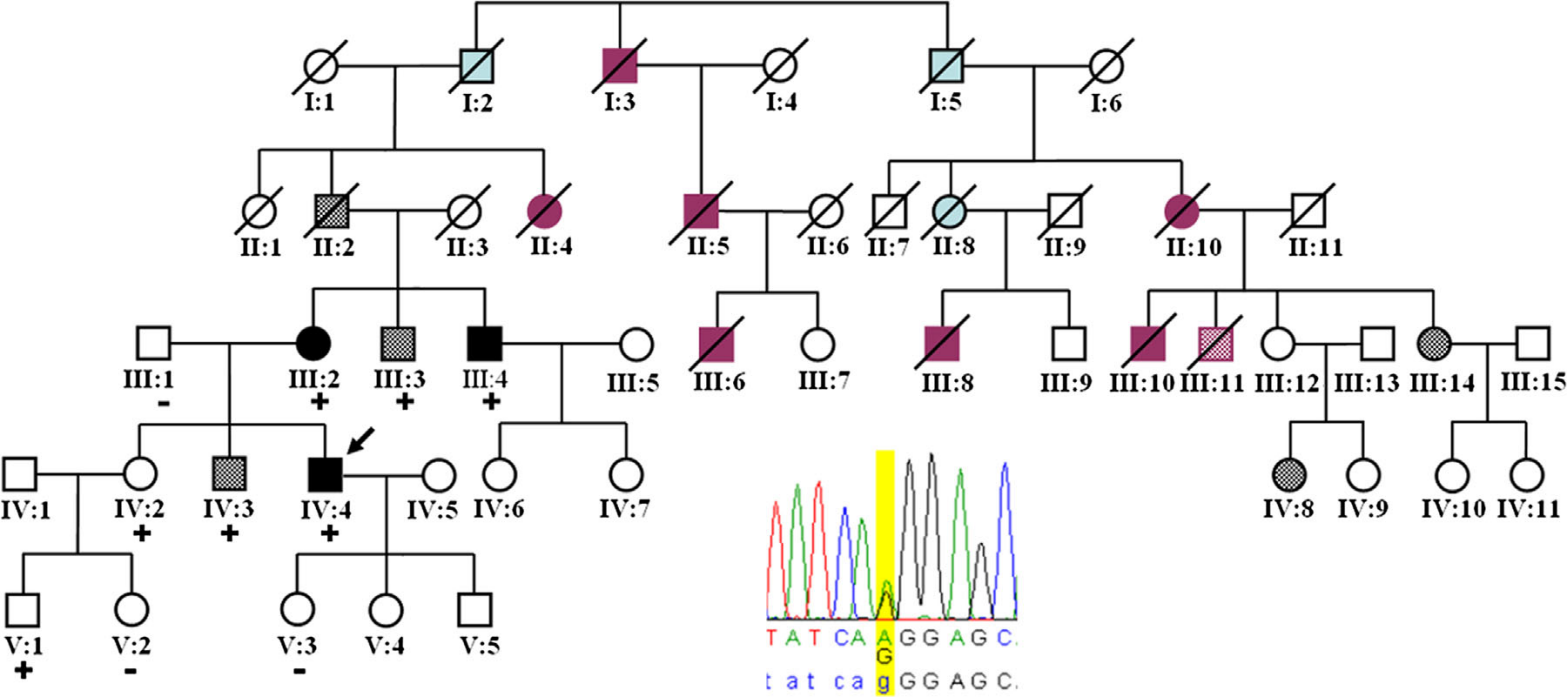
Pedigree of the family. Black and purple filled symbols indicate family members with bilateral hearing impairment, light blue symbols individuals with an unclear hearing status. Approximate time of onset is denoted by black color, representing middle to late adulthood onset, and purple color, representing childhood onset. Completely filled symbols indicate severe hearing loss and squared symbols mild to moderate hearing loss for each onset group. Individuals who were tested positively and negatively for the heterozygous *GRHL2* mutation are indicated by a “+” or “−”, respectively. The index patient IV:4 is marked by an arrow. His 44-year-old sister IV:2 who also carries the mutation has normal hearing thresholds with exceptions at higher frequencies in the right ear. The inset shows the sequence chromatogram of parts of *GRHL2* intron 9 and exon 10 of our index patient, containing the c.1258-1G>A substitution (highlighted in yellow).

The index patient (IV:4) developed type I diabetes at the age of 10 and bilateral progressive hearing loss at the age of 32. He does not show any syndromic features and maintains an academic position. The family history allowed for the tracing back of hearing status over five generations (Fig. 1). Ten family members representing the last three generations were available for genetic analysis.

The hearing loss in the family members with detailed clinical examination is characterized as bilateral and progressive, usually beginning in the fifth decade of life (III:2, III:4, and IV:3) with the earliest documented age of diagnosis being 32 years of age in the index patient (IV:4) and latest diagnosis at age 65 (III:3). However notably, several more distantly related individuals without detailed clinical records (I:3, II:4, II:5, II:10, III:6, III:8, III:10, and III:11) had post-lingual childhood onset reported. Apart from childhood onset hearing loss, III:6 had a reduced IQ and severe epilepsy, which were thought to represent comorbidities contributing to his early death at approximately 40 years of age. Individuals III:8, III:10, and III:11 presented with varying spectrums of communication disorders in addition to childhood onset hearing loss and died prematurely at approximately 50 years of age. III:8 was described as having profound hearing impairment beginning from childhood and had infantile seborrhoeic dermatitis. III:10 was reported as having profound hearing loss that made it nearly impossible to communicate with him. III:11 was able to communicate orally with others, but overcompensated for his hearing loss by utilizing stilted speech.

Although great care was taken to record the hearing statuses of distantly related family members, there were three individuals having ambiguous hearing classifications. I:2 died at approximately 50 years of age at a time when his affection status was not clear. I:5 was reported as being hearing impaired; however, detailed information about onset and severity was unknown. Both these individuals lived in the 19th century, limiting clinical information to what members of the family collected. II:8 had normal hearing early in life but adult onset hearing loss cannot be excluded. III:12 was reported to hear normally, while his daughter IV:8 had mild hearing impairment at approximately 50 years of age. Thus, III:12 may be a non-penetrant mutation carrier. Unfortunately, III:14 and IV:8 were not available for genetic analysis and, thus, we could not test whether the same form of hearing loss segregates in the left and the right side of the pedigree.

### Age of Onset and Progression of Hearing Loss

**Figure 2:**
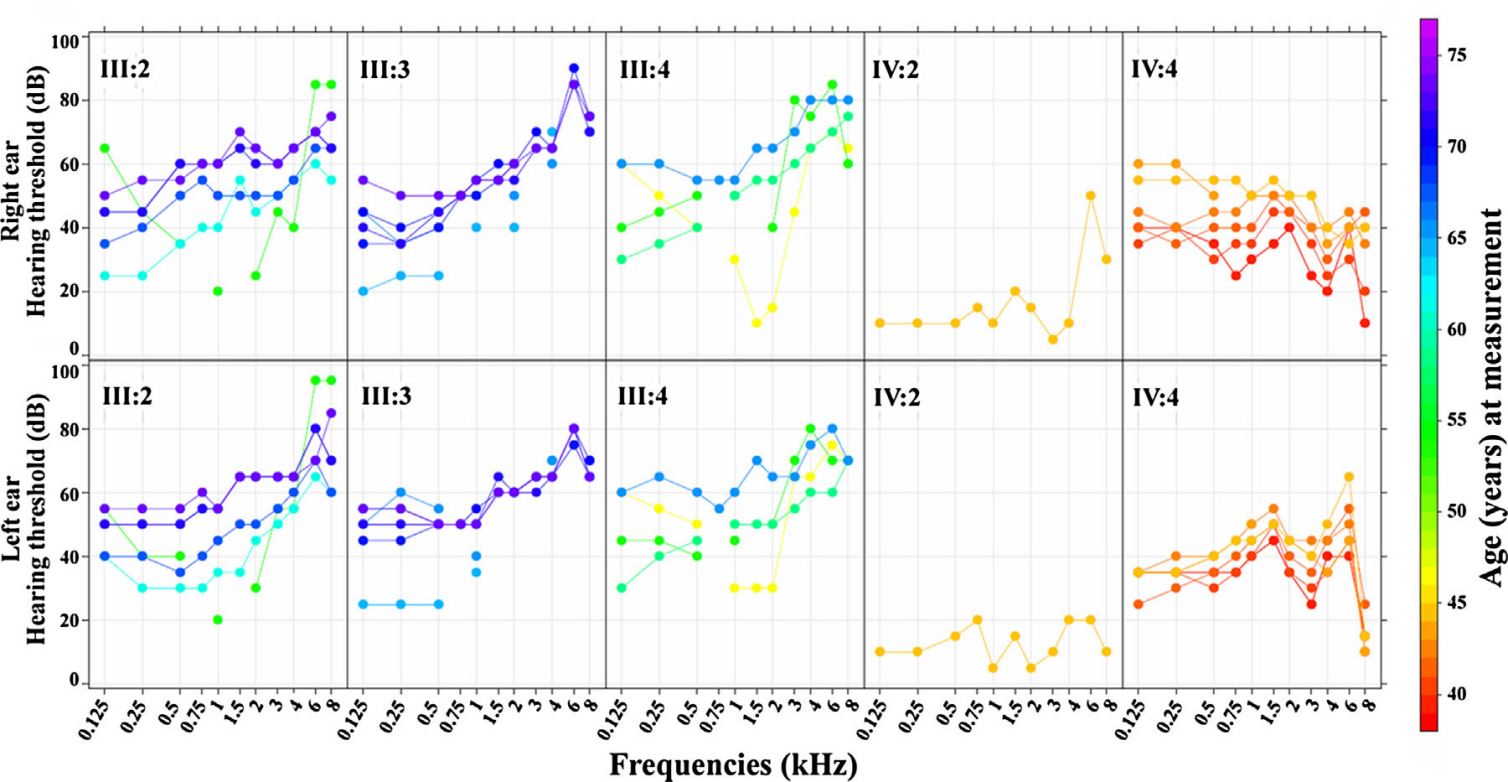
Bilateral pure-tone audiogram data of five mutation heterozygotes (III:2, III:3, III:4, IV:2, and IV:4). The diagrams were constructed with the R Lattice Package [Sarkar, 2008]. The top panels represent measurements of the right ear; the bottom panels of the left ear. Audiograms are scaled by color according to the age at measurement.

Figure 2 shows the bilateral pure-tone air conduction audiograms for family members III:2, III:3; III:4, IV:2, and IV:4. Hearing loss in all frequencies was observed for III:2, III:3, and III:4. Upward sloping profiles in these individuals indicate a greater affection in higher frequencies, particularly at 6 and 8 kHz, as compared to the lower and middle frequencies. IV:2 had only one recorded audiogram from age 44 and had normal hearing thresholds with exceptions at 6 and 8 kHz in the right ear. While IV:4 followed a predictable trend of hearing loss, he displayed an earlier and more severe onset, and was the only affected family member with type I diabetes.

**Figure 3:**
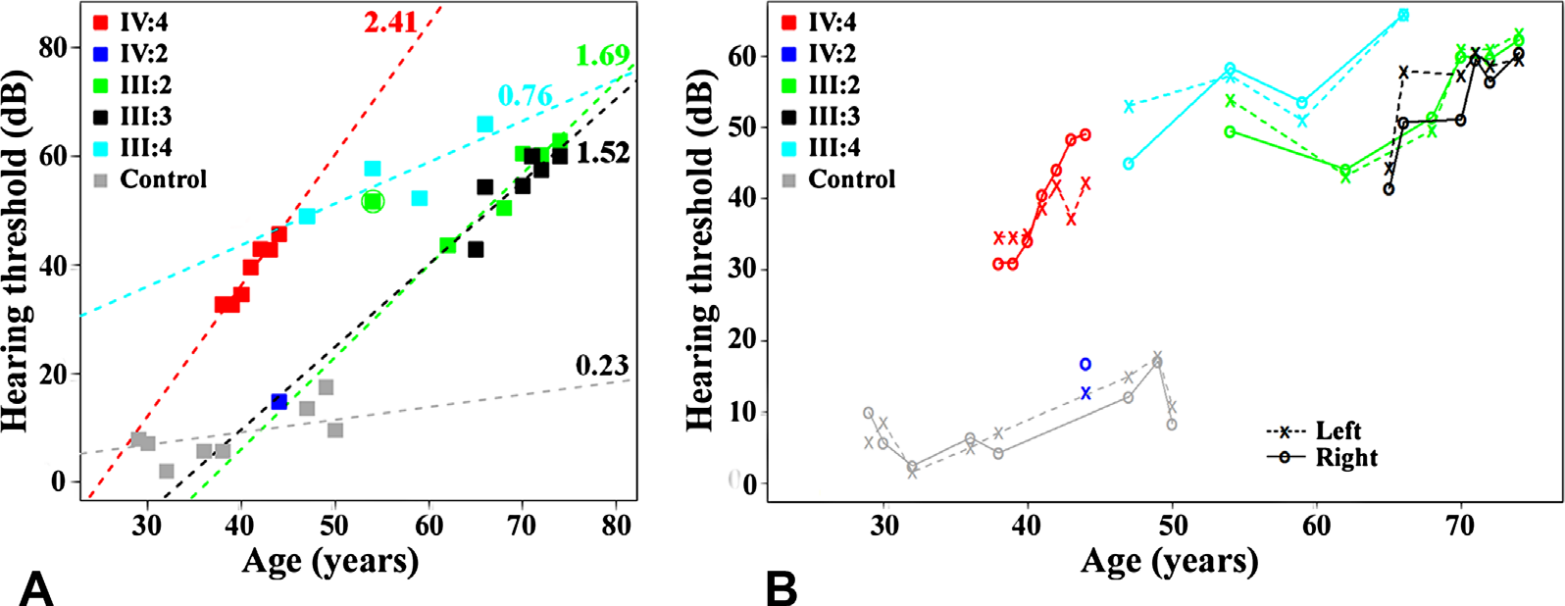
Air conduction pure-tone audiometry analysis from five mutation heterozygotes and one normal hearing control individual. A: The correlation of hearing threshold and age. Linear regression modeling of averaged left and right hearing thresholds across averaged frequencies was performed to obtain ATD values (dB/year) as indicated in the top right portion of each dotted line. An outlier not fitting the linear regression (III:2) is circled and excluded from the statistical analysis. A single plotted square represents one measurement. B: Plot of left and right thresholds (dB) averaged over all frequencies to assess the presence of a left/right difference in hearing loss with increasing age (in years). Each point represents a separate measurement for right (denoted by open circles) and left (denoted by crosses) ears.

Excluding individual IV:2, there was a positive correlation of hearing loss progression and advancing age exceeding what can be expected by normal aging (Fig. 3). We performed a linear regression analysis using the R statistical package [R Development Core Team, 2012] to estimate the progression of hearing loss. Figure 3A shows three of the five mutation carriers clustering linearly, two of which, namely III:2 and III:3, have closely matching annual threshold deterioration (ATD) rates of 1.69 and 1.52 dB/year, respectively, when comparing hearing loss over age after averaging left and right ear thresholds and all frequencies. One outlier measurement was excluded for ATD calculation in III:2 at 54 years of age. Individual III:4 demonstrated greater hearing loss with his initial measurement and had a reduced ATD compared to his other family members with a value of 0.76 dB/year. The index patient IV:4 had an earlier and more severe hearing loss, with an ATD of 2.41 dB/year and when comparing the left and right thresholds averaged across all frequencies, he demonstrated greater hearing loss in his right ear compared to his left as seen in his last three measurements (Fig. 3B). When assessing lateralization of hearing loss using averaged frequencies, we were able to infer from individuals III:2, III:3, and III:4 that there was not a consistent lateral bias between left and right sided hearing loss (Fig. 3B). Apart from IV:2, affected individuals showed a mild (20–40 dB) to moderate (40–55 dB) sensorineural hearing loss in the fourth to seventh decade of life that progressed to moderately severe (55–70 dB) levels in higher frequencies by the seventh and eighth decade.

### Mutation Identification and Characterization

The index patient was negative for mutations in the *GJB2* (OMIM: 121011) gene. He was included in a microarray screen of 50 *GJB2* mutation-negative non-syndromic hearing loss patients, which did not identify any potentially pathogenic copy number variations (data not shown). We then used targeted deafness gene enrichment sequencing (Otogenetics Corporation) to screen for mutations in 80 known deafness genes including 23 DFNA genes, 32 DFNB genes, and 2 DFN genes (with a number of genes being classified as being both dominant and recessive). Syndromic deafness genes were also included. The analysis strategy we employed filtered out apparently non-pathogenic variants, disclosing a single heterozygous c.1258-1G>A substitution in the *GRHL2* gene as pathogenic. The average coverage of *GRHL2* in the analyzed data set was 195x. It is worth emphasizing that mutations in the gene responsible for Wolfram syndrome (*WFS1*), which is characterized by juvenile diabetes mellitus, optic atrophy and progressive hearing loss, were excluded, as *WFS1* is also covered in the NGS deafness panel. To date, we have analyzed 24 additional *GJB2* mutation-negative hearing loss patients and eight normal hearing controls using targeted deafness gene sequencing and did not find any additional mutation in *GRHL2* (data not shown).

Sanger sequencing of genomic DNA (accession: NG_011971.1) showed that the mutation was detected in five family members (III:2, III:3, III:4, IV:3, and IV:4) with middle to late adulthood onset of hearing loss and absent in the normal hearing father (III:1) of the index patient (Fig. 1). Individual IV:2 who was also heterozygous for the *GRHL2* mutation did not report hearing loss at the age of 44. In the youngest generation, we identified one heterozygous individual (V:1) and two individuals (V:2 and V:3) without the mutation. Hearing was normal in all three of these individuals, which was expected, considering their young age.

**Figure 4:**
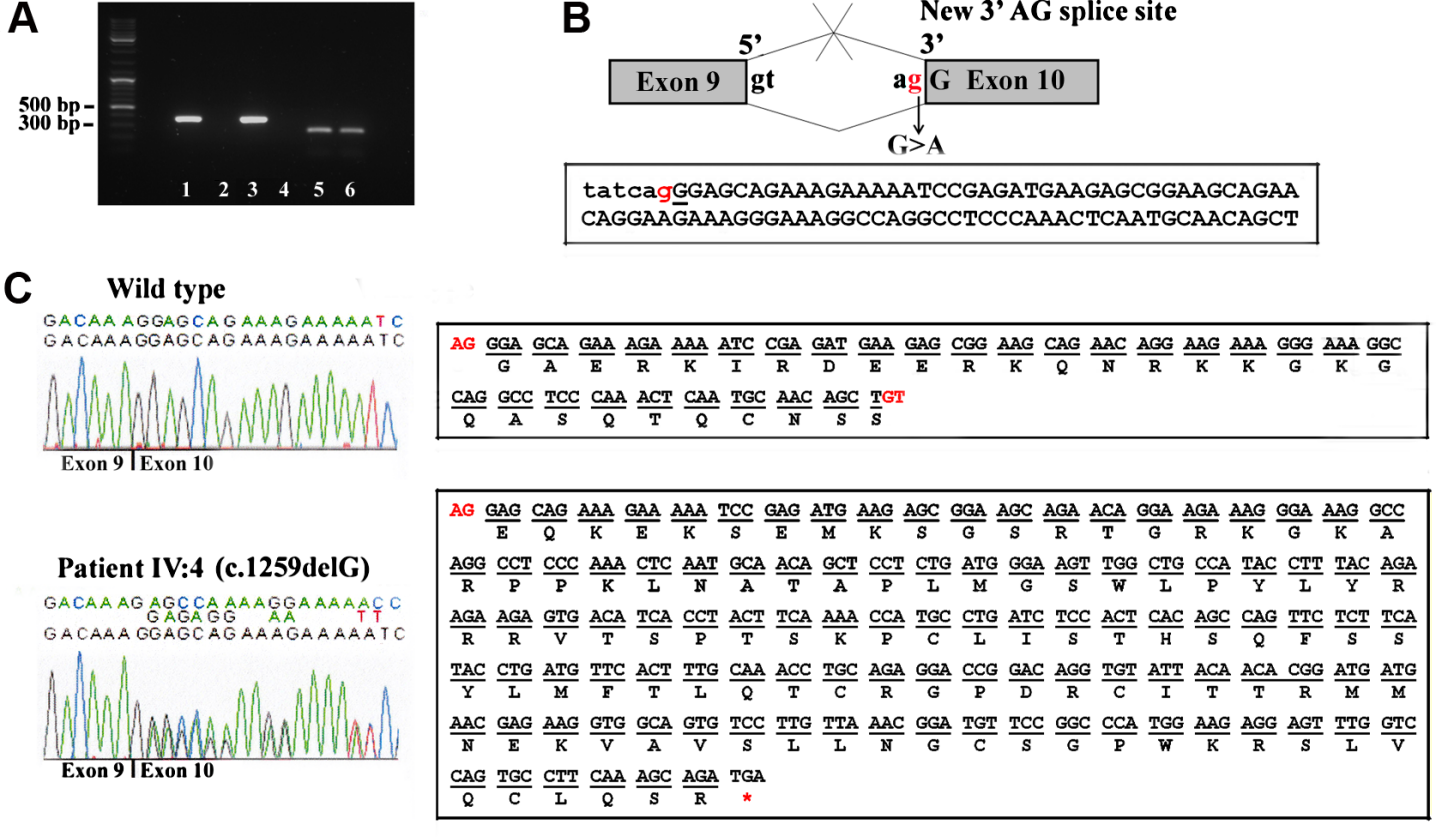
RNA analysis and splice site illustration. A: An agarose gel image shows the RT PCR of the index patient and healthy hearing control cDNA using *GRHL2* primers flanking exons 9 and 11, amplifying a 360 bp product. Human β-actin primers amplified a 250 bp product. Lanes 1, *GRHL2* RT-positive patient cDNA; 2, *GRHL2* RT-negative patient cDNA; 3, *GRHL2* RT-positive control; 4, *GRHL2* RT-negative control; 5, β-actin RT-positive patient; 6, β-actin RT-positive control. B: The *GRHL2* c.1258-1G>A heterozygous mutation introduces a new 3′ AG splice site that causes a deletion in the first nucleotide of exon 10. The splice site is composed of the mutant A and the wild type G in the first position of exon 10. The red nucleotide represents the G>A mutation in intron 9. Partial intron 9 and exon 10 sequence is boxed and depicted in lowercase and capital letters, respectively. The mutated position is highlighted in red and the deleted nucleotide is underlined. C: Chromatograms show cDNA sequencing of the exon 9 and 10-spanning region of interest in the normal hearing control (top) and index patient IV:4 (bottom). The forward cDNA sequence of the index patient shows a heterozygous single nucleotide deletion and a frameshift in exon 10. Encoded amino acid residues are boxed to the right of each chromatogram, with the AG and GT splice recognition sequences depicted in red. A premature stop codon in exon 13 of the index patient is represented with a red asterisk.

We initially predicted that the c.1258-1G>A substitution in the acceptor site of *GRHL2* intron 9 would result in the skipping of exon 10. To test this, we extracted RNA from saliva samples from the index patient and a normal hearing control, synthesized cDNA (accession: NM_024915.3), and amplified a region spanning exons 9 and 11. Comparing the product size of the patient and control through gel electrophoresis, it was demonstrated that the exon 10 was not skipped (Fig. 4A). Instead, Sanger sequencing of the cDNA product showed that a new 3′ AG splice site was shifted by only one nucleotide in the 3′ direction, causing a heterozygous deletion of the first guanine in exon 10 (Fig. 4B). This mutation thus predicts a p.Gly420Glufs*111 in exon 13 (Fig. 4C).

## DISCUSSION

We report on a second DFNA28-causing mutation and the first splice site mutation in *GRHL2* in a family affected with non-syndromic hearing loss. Previously, only one mutation in *GRHL2* has been associated with hearing loss [Peters et al., 2002]. The mutation described here confirms that mutations in *GRHL2* cause postlingual progressive hearing loss. In this light, it may also be worth following up the marginally significant association of presbycusis with *GRHL2* variants [Van Laer et al., 2008], using larger cohorts.

This newly identified *GRHL2* mutation constitutes a type IV nonclassical (intronic) splicing mutation, which could have been misinterpreted as a classic (type I) splice defect if cDNA was not analyzed [Eng et al., 2004]. In the index patient, the heterozygous c.1258-1G>A mutation activates a cryptic 3′ splice site in genomic DNA. The first nucleotide in exon 10 is a G, and the consequence of the G>A mutation is a one nucleotide shift of the splice site consensus sequence resulting in a deletion of the first nucleotide in exon 10 and a frameshift ending in an in-frame stop codon. Given the cDNA sequence data, it is predicted that this mutation negatively impacts the protein. There are several hypothesized outcomes as a consequence of mutations leading to a frameshift and premature termination, namely nonsense-mediated decay, loss of protein function via protein truncation, and alteration of protein-folding kinetics leading to proteolysis [Gregersen et al., 2000; Williams et al., 2003]. Additionally, they may act as dominant-negative mutations [Schell et al., 2002].

The maximum entropy model called MaxEntScan by Yeo and Burge [2004] provides a splice site prediction algorithm that assesses the relative strengths of new splice sites. High MaxENT scores indicate an increased efficiency in splicing. When considering the impact of a mutation in a splice consensus region on exon inclusion or exclusion, the contributions of both the 3′ and 5′ splice site MaxENT scores should be taken into consideration [Shepard et al., 2011]. The wild type *GRHL2* exon 10 MaxENT score of the 3′ site is 10.1 and the 5′ is 7.4. The cryptic 3′ splice site generated by the c.1258-1G>A mutation has a strong MaxENT score of 9.5. A 6% decrease compared to the wild-type is most likely not sufficient to cause exon skipping or other aberrant splicing, especially since MaxENT scores become increasingly negative as splicing becomes less likely to occur. This further substantiates our cDNA sequence analysis.

GRHL2 participates in the differentiation and maintenance of epithelial cells throughout life [Werth et al., 16]. Impaired epithelial cell integrity is the most reasonable pathological explanation as to its involvement in late-onset hearing impairment [Peters et al., 2002; Van Laer et al., 2008]. Considering a number of factors that are useful for predicting haploinsufficiency such as temporal expression, proximity to other haploinsufficiency genes, interaction partners, and genetic implication in disease [Huang et al., 2010], *GRHL2* is predicted to have a high probability of exhibiting haploinsufficiency. It is plausible to assume that the hearing loss in the present family and the previously reported family [Peters et al., 2002] is due to *GRHL2* haploinsufficiency.

The results show that the heterozygotes for the c.1258-1G>A mutation in *GRHL2* have progressive, bilateral hearing loss with a typical onset in middle to late adulthood. The variability in the onset of hearing loss and audiometric profiles in heterozygotes argue for the interplay of other genetic or environmental factors in determining the events leading to hearing loss. Comorbidities independent of hearing loss such as epilepsy, reduced IQ, and type I diabetes may influence the onset and severity of hearing loss and explain this variation. Alternatively, given the enormous genetic heterogeneity of hearing loss and the high rate of marriage among hearing-impaired individuals, it is possible that the family members with a documented childhood onset in the right branch of the pedigree, who were not available for genetic diagnostics and detailed clinical examination, suffer from a distinct form of dominant deafness.
